# HOBE+, a case study: a virtual community of practice to support innovation in primary care in Basque Public Health Service

**DOI:** 10.1186/1471-2296-14-168

**Published:** 2013-11-05

**Authors:** Galder Abos Mendizabal, Roberto Nuño Solinís, Irune Zaballa González

**Affiliations:** 1 O + berri, Instituto Vasco de Innovación Sanitaria (The Basque Institute for Healthcare Innovation), Plaza de Asua n 1, Sondika, Bizkaia 48150, Spain

**Keywords:** Basque Country, Primary care, Innovation, CoP, Online professional community of practice, HOBE+, Osakidetza, O + berri

## Abstract

**Background:**

A virtual professional community of practice (VCoP), HOBE+, has been set up to foster and facilitate innovation in primary care. It is aimed at all primary care professionals of the Basque Public Health Service (Osakidetza) in the provinces of Biscay and Araba. HOBE + is a VCoP that incorporates innovation management from the generation of ideas to their implementation in primary care practice.

**Methods:**

We used a case study method, based on the data provided by the technology platform that supports the VCoP, and from a survey completed by HOBE + users. The target population was all primary care staff (including all professional categories) from Araba and Biscay provinces of the Basque Country (Spain), who represent the target users of the VCoP.

**Results:**

From a total of 5190 professionals across all the professional categories invited to join, 1627 (31.3%) actually registered in the VCoP and, during the study period, 90 (5.5% of the registered users) participated actively in some way. The total number of ideas proposed by the registered users was 133. Of these, 23 ideas (17.2%) are being implemented. Finally, 80% of the users who answered the satisfaction survey about their experience with HOBE + considered the initiative useful in order to achieve continuous improvement and real innovation in clinical and managerial processes.

**Conclusions:**

The experience shows that it is possible to create a virtual CoP for innovation in primary care where professionals from different professional categories propose ideas for innovation that are ultimately implemented.

This manuscript objectives are to assess the process of developing and implementing a VCoP open to all primary care professionals in Osakidetza, including the take-up, participation and use of this VCoP in the first 15 months after its launch in October 2011. In addition, the usefulness of the VCoP was assessed through a survey gathering the opinions of the professionals involved.

## Background

### Open innovation and knowledge management

Sustainability in competitive environments requires companies to share knowledge beyond their traditional boundaries in order to innovate [1]. This open innovation paradigm (open innovation being a term coined by Professor Henry Chesbrough [2]), postulates the need for internal and external flows of knowledge around organisations to extract the most value from their innovative potential.

In the healthcare sector, even in countries with universal coverage, tax funding and quasi-monopoly of public providers (like the Spanish National Health Service), the pressing problems of chronic diseases and multimorbidity, as well as rapid technological and social changes, jeopardise the sustainability of healthcare systems. Innovation is regarded as critical for long-term sustainability in this sector.

The discipline known as knowledge management deals with the study of the most important decisions about knowledge itself, and has become one of the most common approaches in the field of strategic management [3,4]. Knowledge management doesn’t have a single definition. It can be used to understand performance in organisations. Being healthcare delivery a knowledge driven process, knowledge management provides an opportunity for improvement and innovation in processes. A review on this discipline in the healthcare industry shows important insights [5] and states that knowledge management is systemically more complex in healthcare due to existing tensions within and between issues in three domains: specific value-laden aspects of clinical practice; normalization of workplace practice into generic process flows; and the technical integration of disparate information systems that is key for knowledge application.

The content of the discipline includes the analysis of knowledge management processes (development, integration, protection, transfer and exploitation) needed to get the most value from efforts to generate intellectual capital in organisations. More specifically, Sáez Vacas et al. [6] define it as the process of identifying, grouping, sorting and continually sharing knowledge of all kinds to meet present and future needs to identify and exploit knowledge (both pre-existing and newly acquired) in order to develop new opportunities.

The knowledge creation spiral model of Nonaka and Takeuchi [7] integrates open innovation and knowledge management. These authors argue that an organisation cannot create value without the initiative of different individuals and interactions that are established in the working groups. Further, they indicate the existence of cross-organisational knowledge from relationships between organisations and external agents, whose existence adds value to the open innovation process. These authors describe the knowledge creation spiral this way. First of all, they divide the knowledge in two types:

*Explicit knowledge*

Knowledge that can be structured, stored and distributed. Such knowledge can be transmitted easily from person to person.

*Tacit knowledge*

Those skills that are part of our mental model, the result of our personal experience and involves intangible factors such as beliefs, values, insights, intuition, etc … , and therefore can not be structured, store or distribute.

Thus, the explicit knowledge can be easily processed by a computer, distributed electronically (eg via e-mail) or stored in a database, however, the nature of tacit knowledge complicates it’s processing and distribution. This requires transforming it into concepts that everyone can understand, and this way, convert it into explicit knowledge (and the other way around).

So, both open innovation and knowledge management require specific tools to facilitate the flow of ideas in a structured and standardised way in order to achieve specific results.In this context, communities of practice (from now on CoPs, as they are commonly known in the literature) have been associated with knowledge management as people have begun to see them as a way to develop social capital, nurturing new knowledge, stimulating innovation, and sharing tacit knowledge within an organisation.

### Communities of practice and virtual communities of practice

Advances in information technologies over the past 10 years have allowed the general public and professionals to obtain easy access to information from diverse areas of knowledge. More specifically, Web 2.0 applications enable the exchange of knowledge and opinions between different users, allowing a multidirectional flow of information; therefore, they facilitate the creation of virtual communities.

According to Wenger, McDermott and Snyder [8-12], a CoP is a group of people who share a concern, a set of problems or a common interest in a topic at a personal or professional level, and who increase their knowledge and experience in this area through continued interaction. CoPs can become formidable tools for managing knowledge in organisations beyond the limits of formal systems. Besides their benefits in terms of dissemination of organisational knowledge, such communities can also serve as talent integration tools and help to strengthen the sense of belonging to an organisation.

A CoP can be a very effective tool for knowledge exchange between peers and between different hierarchical levels. Indeed, established hierarchies tend to disappear as people become focused on the specific knowledge area or topic itself. Habitually, a facilitator (also called moderator or coordinator depending on the authors) is needed in order to galvanise and manage the community. However, self-regulation should be allowed and there should be no manipulative authoritarian attitudes, rather members should be stimulated with questions, and proposals for improvements and actions, together with active networking.

The CoP concept has been taken forward from an analytical or theoretical approach to a management tool that can be deliberately cultivated [11]. In particular, Wenger, McDermott and Snyder [12] focus on the use of CoP as a knowledge management tool. The authors suggest that organisations can improve the capacity of their members through the use of this type of community.

While the concept is subject to different interpretations [13], we can identify the main characteristics of a CoP, namely the support for formal and informal interaction among users to promote the exchange of knowledge and encourage learning. According to the work of Wenger and colleagues [12], CoP’s are characterised by three features: a domain of knowledge, a community of people interested in this area of knowledge and shared practice within that scope.

Systematic reviews of the field show that CoP-related publications have focused on areas such as education or business, significantly less having been published on their use in the healthcare sector [14,15]. As for evidence of the effectiveness of CoPs in healthcare, there have been some reports of positive effects on continuing education, knowledge transfer and adoption of innovation [14-19]. However, these studies are qualitative and very heterogeneous, and the CoPs evaluated have been developed in the context of complex and multifaceted interventions. These factors make it difficult to attribute specific effects to the CoP or to draw strong conclusions.

One of the key factors in the success of CoPs is the facilitator, who has a crucial role to play in ensuring the effective functioning of the CoP, especially in the case of a virtual CoP (hereinafter VCoP). The facilitator’s mission is to promote participation and manage the content exchanged between members of the CoP, identifying relevant content and storing it properly for easy retrieval.

Some consider VCoPs to be "semi-communities" as, being computer-mediated contact, they lack some of the most important aspects of communication. This has been changing in the last decade, however, due to advances in social software tools. There are platforms where you can communicate in written, spoken and even "symbolic" ways, such as Second Life and other virtual world environments.

In VCoPs, peripheral participation refers to people who do not send messages and/or do not contribute to the forums, but do connect and read what is said in the debates. These users are known as "lurkers" and even though they do not contribute directly they usually obtain benefits from the knowledge sharing that takes place on the platform, applying ideas and improvements in their day-to-day job. These types of benefits are, however, difficult to quantify.

### The HOBE + VCoP

HOBE + (Hobe, derived from the Basque word for improvement, “hobekuntza”) is an innovative online VCoP of primary care professionals developed in order to generate, identify and promote innovation and improvement within the Basque Health System. Launched in October 2011 by four primary districts of Osakidetza in Biscay and the Basque Institute for Healthcare Innovation (O + berri), Hobe + provides an opportunity for primary care professionals to identify, propose, define and develop innovative ideas that arise in their daily work in Osakidetza. This initiative encompasses a process of innovation management from idea generation to its eventual implementation. In June 2012, the Araba primary care district joined the platform.

Participation in HOBE + is voluntary. All primary care workers from Biscay and Araba are invited to use the platform, but it is their own decision whether to participate or not. HOBE + users can share their innovative ideas or suggestions for improvements, as well as access those proposed by others, at different levels of detail. Once ideas have been introduced, users are able to discuss them, enriching the ideas and offering alternatives through their comments.

In previous reviews [14-19], we found no publications on VCoPs specifically dedicated to innovation in primary care. Therefore, the objectives of the present paper were to assess the process of developing and implementing a VCoP open to all primary care professionals in Osakidetza, exploring the take-up, participation and use of this CoP in the first 15 months after its launch, and to assess the opinions of the professionals involved in this initiative.

## Methods

We adopted a case study approach [20] based on researchers’ observations, data provided by the technology platform supporting the VCoP, and data from a survey completed by HOBE + users.

Regarding the survey, the 1,627 registered users of HOBE + were consulted. A survey was sent out on 10 and 11 December 2012 to gather extra information about the users profile, usage and opinion about HOBE+.

### Variables assessed

First, data were collected on the processes involved in the creation and implementation of the HOBE + VCoP based on observations made by the researchers during the course of the project.

Second, the dashboard of the technology platform offers quantitative data on how primary care professionals take-up, use and participate in the VCoP. The following variables were explored:

● Acceptance of the VCoP by primary care professionals, measured in terms of the total number of professionals that take-up the offer and register in the VCoP.

● Participation, as measured by the rate of interactions between professionals across the VCoP, in terms of the numbers of contributions and comments.

● Use, as measured by the number of entries in the VCoP, the number of ideas put forward, and the number of comments posted.

● Impact, as measured by the number of ideas carried through to implementation.

● Third, from the survey we analysed the following variables:

● User profile information

● Perception of the usefulness of HOBE+

These data come from the 233 users who completed the survey within 15 days of receipt, the response rate being nearly 15% (233 of the 1627 registered users).

## Results and discussion

## Results

### Creation of working groups to manage the VCoP

**Figure 1:**
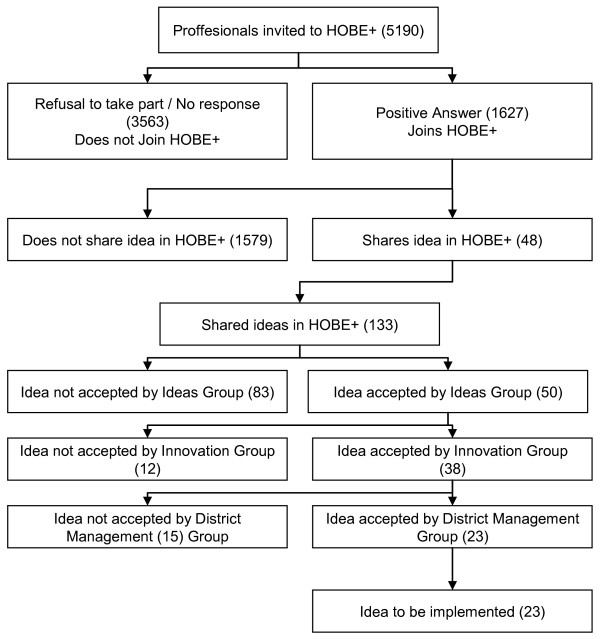
**Innovation flow chart.** The development, implementation and use of a CoP for Innovation in Primary Care.

An innovation process was established in order to detect and refine innovation ideas in HOBE+. This process defines the potential channel for ideas, from their proposal to their eventual implementation in primary care organisations. Figure 1 summarises the processes involved including the management of the proposals by the various agents and working groups. For this management of the actions during each phase of the innovation process, two working groups were created: the Ideas Group and the Innovation Group, both made up of non-managerial staff from various levels of the organisations participating in HOBE+. Further, the CoP innovation process was defined in order to manage platform usage and the work of these groups. Both groups are facilitated by O + berri.

The Ideas Group is responsible for the initial screening of ideas and galvanisation of the platform. Its members filter existing ideas, fleshing out each selected idea in sufficient detail and depth to ensure they could become real projects or improvements. This is achieved by galvanizing the platform, fostering debate and seeking information that could support ideas that emerge. Once an idea is solid enough, Ideas Group members decide whether they can foster its implementation based on its complexity and the possible need for further validation from other hierarchical layers. If further steps are needed, selected ideas are passed to the Innovation group. Hence, the main duty of the Ideas Group members is to serve as a first filter to ideas and, where possible, supply background information such that they could be selected as projects. Ideas group members dedicate around 16 hours a month to the community, focused mainly on these tasks.

The Innovation Group carries out a more in-depth analysis, as well as studying the feasibility of the proposals coming from Ideas Group. In most cases, members of this group are more senior and experienced than the Ideas Group members and have enough background or influence at a managerial level for validating or filtering out ideas. At the same time, they refine the associated proposals and decide whether they could be implemented from this level or they need approval from the Management Group.

The Management Group, also facilitated by O + berri, has the ultimate responsibility for taking decisions on proposed changes, improvements or initiatives, as well as integrating them into management plans or passing them on to other levels of management. It is also responsible for identifying the corresponding resources and the development of innovation projects that require prior research or pilot schemes.

O + berri’s facilitator role includes the coordination of the 3 working groups, preparation and conduction of the meetings of each group and supervision, follow up and support in the development of every selected idea, At the same time, if one of the ideas approved by the Managers group needs any special coordination or agreement with Osakidetza’s Headquarters for it’s implementation. Therefore O + berri plays the coordinator role between Osakidetza Headquarters and HOBE + member groups.

### Technology platform

In order to identify a suitable IT platform for HOBE+, several national and international healthcare-related CoP projects and tools were analysed (see Appendix 1). Among all these options, we decided to purchase the licence for the IdeaScale software to support the CoP based on its flexibility to be adapted to the requirements and functionality required for the innovation process. This software offers extensive leverage of the database generated, and provides administrators with valuable information to assess how the CoP is being used, participation rates and results, as well as the status of the ideas generated by users.

### Launch and development of the HOBE + innovation community

On 6 October 2011, HOBE + was launched. That day, all primary care professionals in Biscay with an email address received an invitation to join the community. To sign up, all they had to do was click the link in the email and set a password.

In June 2012, Araba primary care district decided to join HOBE + not only to develop an innovation process in their own district but also to be able to share knowledge and best practices with other primary care districts in Osakidetza.

### Acceptance, participation, use and impact

Acceptance was assessed in terms of the number of professionals who registered in the HOBE + VCoP out of the total number who received an invitation.

In Table 1 we present the total number of professionals registered as well as the percentage registered by professional category.

**Table 1:** Acceptance of the CoP by the primary care professionals (total and by category)

**Acceptance of the CoP by the primary care professionals**	**n (%)**
Professionals invited	5190 (100)
Doctors Invited	1732 (33)
Nursing professionals invited	1916 (37)
Administrative staff invited	1058 (20)
Technicians invited	128 (3)
Other staff invited (unspecified)	356 (7)
Professionals registered (positive answer to invitation over invited professionals)	1627 (31)
Doctors registered (among registered professionals)	520 (32)
Nursing professionals registered (among registered professionals)	486 (30)
Administrative staff registered (among registered professionals)	340 (21)
Technicians registered (among registered professionals)	95 (6)
Other staff registered (unspecified) (among registered professionals)	186 (11)

In terms of participation, Table 2 shows the proportion of “real”, that is, active, users of the platform compared to those that only use HOBE + to obtain information created by other users.

**Table 2:** Participation in the CoP (among registered users)

**Participation in the CoP**	**n (% of the total number of registered users)**
Registered users that have contributed ideas	58 (4)
Registered users that have commented on ideas	99 (6)
**Idea contributors by professional category**	**n (% of the total number of idea contributors)**
Doctors	23 (40)
Nursing professionals	23 (40)
Administrative staff	9 (15)
Technicians	2 (3)
Other staff (unspecified)	1 (2)
**Comments to ideas by professional category**	**n (% of the total number of comments)**
Doctors	39 (39)
Nursing professionals	31 (32)
Administrative staff	18 (18)
Technicians	7 (7)
Other staff (unspecified)	4 (4)

Table 3 reports data regarding the use of the VCoP. It lists the total number of activities registered in HOBE+, including all the ideas submitted and the actions that they generated, such as comments or votes received.

**Table 3:** Use of the VCoP

**Use of the VCoP**	**n**
Total number of ideas submitted	133
Total number of comments regarding ideas	916
Total number of votes (positive or negative) on the ideas	2013
Average number of comments per idea	6.3
Average number of votes (positive or negative) per idea	16.4
Total number of visits	16,355
Total number of pages visited	77,378
Average length of visit (min:sec)	06:44
Average number of pages/visit	4.73

The impact that the HOBE + VCoP had had by the end of its first 15 months of activity is reflected in Table 4. Of a total of 133 ideas suggested, 23 were selected by the Management Group for implementation. These cover a wide variety of topics, from implementing specific improvements to the electronic medical record, improving stock management in healthcare centres, and designing new centralised purchasing procedures for specific items, to developing initiatives to promote training and information for adolescents and young people in key aspects of health: lifestyle, sexuality, socialising and mental health and so on.

**Table 4:** Results in terms of ideas taken forward

**Results in terms of numbers of ideas taken forward**	**n (%)**
Total ideas submitted	133 (100.00)
Ideas accepted by the Ideas Group	50 (37.59)
Ideas accepted by the Innovation Group	38 (28.57)
Ideas accepted by the Management Group	23 (17.29)
Ideas ultimately implemented or in the process of being implemented	23 (17.29)

Qualitative analysis of the ideas submitted to date shows that they tend to be related to the improvement of current clinical and managerial processes, IT problems and day-to-day situations, rather than disruptive innovations.

Additionally, O + berri sent a survey to registered users of the platform on 10 and 11 December 2012 in order to gather extra information about the user profiles and opinions of HOBE+. The target population for the survey were the 1,627 registered users of HOBE+. The data analysed in this study are from the 233 users who completed the survey within 15 days of receipt, meaning that the response rate was nearly 15% (233 of the 1,627 registered users).

Based on this survey, Table 5 shows the extra information obtained related to the profile of HOBE + users.

**Table 5:** User demographics and platform usage metrics

**User distribution by primary care district**	**Survey respondents** **n (%)**	**Invited professionals** **n (%)**
Bilbao primary care district	88 (37.76)	1179 (22.7)
Ezkerralde - Enkarterri primary care district	56 (24.03)	1076 (20.7)
Araba primary care district	31 (13.30)	999 (19.3)
Uribe primary care district	30 (12.87)	734 (14.1)
Interior primary care district	28 (12.01)	1202 (23.2)
**User distribution by sex**		
Men	53 (22.75)	1137 (21.9)
Women	180 (77.25)	4053 (78.1)
**User distribution by age**		
50-69 years	128 (54.93)	No Data
35-49 years	93 (39.91)	No Data
20-34 years	12 (5.15)	No Data
**User distribution by years working in Osakidetza**		
More than 20 years	147 (63.10)	No Data
10-19 years	61 (26.18)	No Data
0-9 years	25 (10.72)	No Data
**User frequency of accessing HOBE+**		
Several times a week	47 (20.17)	N/A
Once a week	106 (45.49)	N/A
Once a month	54 (23.17)	N/A
Only registered	26 (11.15)	N/A

Additionally, Table 6 shows data related with the user perception of the usefulness of various aspects of the platform.

**Table 6:** Perceived usefulness of HOBE+

	**Totally agree** **n (%)**	**Agree** **n (%)**	**Partially agree** **n (%)**	**Disagree** **n (%)**	**Totally Disagree** **n (%)**
Does HOBE + offer opportunities to increase users’ knowledge thanks to the vision of other users and / or experiences in other districts?	35 (15.02)	98 (42.06)	72 (30.90)	26 (11.15)	2 (0.85)
Does HOBE + offer users opportunities to establish new professional contacts both within and outside the platform that could be beneficial for their day-to-day activities?	18 (7.72)	67 (28.15)	52 (22.31)	80 (34.33)	16 (6.86)
Does participation in the HOBE + community provide direct support with the resolution of day-to-day problems?	58 (24.89)	48 (20.60)	81 (34.76)	42 (18.02)	4 (1.71)
Is HOBE + a useful tool for detecting and implementing innovations and improvements?	40 (17.16)	80 (34.33)	67 (28.75)	32 (13.73)	14 (6.00)

## Discussion

This case study offers an overview of the main aspects of setting up and developing a virtual community of practice for innovation in primary care in Biscay and Araba, as well as the results of its activity at the end of its first 15 months of life.

HOBE + serves as a practical example of the implementation of an initiative based on evidence in the literature on CoPs and open innovation. The openness to professionals of the five organisations involved, irrespective of their professional category, is a key characteristic of the initiative. The acceptance and participation in HOBE + seems satisfactory, outnumbering rates reported for other similar projects in the healthcare sector [15,18]. The professional category most well represented in HOBE + is family doctors, although nurses and administrative staff have also taken part in significant numbers. In terms of participation, however, a subset of users is notably more active. Specifically, 96.4% of those who signed up did not take an active part in HOBE+; therefore, to date, ideas have been created and defined by the other 3.6% of the registered users.

Such figures are consistent with other data available on participation in CoPs [16,21]. Three levels of participation are usually observed. The first consists of a hard core of individuals who are very active in the community, the leaders. This group is usually small and does not make up more than 10 to 15% of the community. Then, there are active members who regularly participate in meetings and online discussions, but without the regularity or intensity of the leaders. This group is also small and usually represents 15 to 20% of the members. Finally, most members of CoPs are on the periphery and not actively involved in community activities. The important point here is the legitimacy of peripheral participation, i.e., when the learner hears and reads, but does not say or write anything. Traditionally, this type of activity is not considered to be participation and is discouraged, while in contrast, in the theory of CoP it is an essential part of the learning process.

Based on the results from the survey (Table 6) and informal input from various registered but non-active healthcare professionals, there is evidence that non-active users do consult the platform and take advantage of the knowledge sharing taking place through HOBE+.

Indeed, web metrics (Table 3) show great activity on the platform during these months, in terms of both number of visits and pages visited, that supports the view that the platform is utilised by more people than the 3.6% of active users. It also shows a significant average time per visit which reinforces the perception of usefulness found in the survey.

Further, once the ideas that emerge are implemented they potentially have an impact on all the organisations involved in HOBE+, directly affecting the healthcare professionals in all centres in one way or another, depending on the nature of each idea. That is, even though the main contributors to HOBE + correspond to just 3.6% of the registered users, a much larger number of users reap direct benefits from the knowledge, best practices and innovations discussed.

These findings contrast to some extent with the conclusions of Kislov and colleagues [22] that the construction of CoPs from scratch in primary care in the UK was problematic; yet they do agree that the multidisciplinary nature of the CoP is not a limitation. On the other hand, although there is some common ground in the area of knowledge, comparisons are difficult given the contexts, approaches and instruments used in the studies.

The establishment of an innovation process strengthened by the creation of support groups helped with the definition and galvanisation of ideas and, ultimately, led to the implementation of 23. In the absence of benchmarks, we cannot assess whether this number is high or low. The support of these selected 23 initiatives by the Management Group was decisive in making doable what seemed inconceivable some months ago, as all of the ideas involved cross-organisational issues that affected all the organisations and to be taken forward needed official support, requiring negotiation with Osakidetza headquarters. Some of these 23 ideas reflected changes that had been called for by primary care professionals in Osakidetza in the past and that only now, via the structured process of HOBE + and the efforts of its working groups, are finally being implemented or scheduled for implementation in approved action plans for the coming months.

Regarding user perception, it should be highlighted that 80% of the survey respondents rated the initiative as useful to achieve continuous improvement and real innovation in Osakidetza.

At the same time, 80% of HOBE + users felt that the organisation takes into account and generally supports initiatives launched in the community, implying an alignment between different organisational levels. This should help provide professionals of the organisations involved in HOBE + with a sense of empowerment and greater levels of autonomy and responsibility. These factors make it possible for certain improvements and innovations (those that are not highly complex and require only a limited level of responsibility) to be defined and implemented without having to wait for approval from supervisors for every step.

The present study confirms the conceptual framework of the successful factors for VCoPs proposed by Probst and Borzillo [21] and, moreover, their applicability in the healthcare sector: the presence of facilitators and leaders that offer support is essential to the success of a CoP, as is the availability of a wide and multidisciplinary base of potential participants. Moreover, the CoP must have clear objectives, operate in an enabling environment that will generate trust, the technology platform must be user-friendly and there must be transparency and feedback to participants about their ideas. HOBE + appears to satisfy these requirements, which would explain its initial success and bode well for its future sustainability.

The facilitator role played by a "neutral" agent (in this case O + berri) has been key in facilitating the debate between professionals and ensuring that every single organisational level involved in the development, support and implementation of the idea assumes their responsibility during the implementation process. Indeed, except in specific cases, the work of the facilitator from the identification of the idea to its implementation has been absolutely necessary to prevent initiatives from grinding to a halt. Moreover, as Probst and Borzillo [21] proposed, there are several advantages of grouping managerial sponsors and COP coordinators into the same committee in order to achieve a more effective governance of the innovation process in which that managerial sponsors can assess COP’s activity with a complete overview of the value of the different proposals.

Although there is a defined work process, the active participation and identification of ideas in reality involves a few particularly motivated users contributing on a voluntary basis (the 3.6% of registered users mentioned above) [23]. The present study does not address the theoretical debate about whether CoPs [24,25] only stimulate incremental innovation, while restricting disruptive innovation as there is no hybridisation of knowledge (e.g., from outside the healthcare sector).

On the other hand, the opening of HOBE + to other agents such as hospital staff, patients and other stakeholders outside healthcare has been debated since the beginning of the project. This is a complex issue, the current dominant view being that extending the field of knowledge of the CoP has to be balanced against the risk of losing a sense of belonging and shared practice among the professional community.

Finally, this study has the limitations associated with a descriptive approach and lacks comparability in many respects. In particular, caution is required in the interpretation and extrapolation of the results as we have not identified comparable projects in primary care anywhere else in the world.

Another relevant limitation is the lack of an economic evaluation of the value of the implemented ideas versus the cost of their development and implementation. Most of the ideas implemented are related with business processes refinements and time savings. The ones that are in process of being implemented are related with logistics management and inventory improvements, requiring more time for their full extension and impact assessment. The impact assessment of all these 23 ideas is definitely an interesting topic for a future new paper regarding the cost-benefit analysis of HOBE+.

At this preliminary stage of development, the user’s perceived usefulness of the experience is positive in the 80.24% of the cases, based on the survey respondents data.

This provides a clue of the perception of the HOBE + users; however, as mentioned before, at this stage we can not evaluate detected improvements impact.

## Conclusions

HOBE + illustrates that it is feasible to create a CoP for innovation and improvement in primary care to which all staff can contribute, irrespective of their professional status.

In addition to the identification of new ideas, HOBE + offers other benefits such as the exchange of pre-existing knowledge and best practices between peers. This is a clear example of the added benefits that both active and not active members (the "lurkers" mentioned earlier) can reap from this type of community, helping them to improve their performance and daily practice.

Therefore, the benefits and range of improvements achieved in Basque primary care through HOBE + should not only be measured quantitatively in terms of the number of new ideas, but also qualitatively considering the impact of the knowledge exchange and discussion among peers, as can be seen from the results of the survey.

Last but not least, the facilitator role has been critical in ensuring the progress of ideas along the innovation process (from their detection, definition and development, to implementation) including the coordination of three support groups (focused on Ideas, Innovation and Management).

To sum up, the introduction of new innovations is possible in Osakidetza with initiatives like HOBE+, but their sustainability over time will depend on the generation of a culture innovation that ensures the continuous detection, development and implementation of new initiatives, increasing the responsibility and the active role of primary care professionals in a natural way, making the progress of ideas less dependent on (and ultimately independent of) the intervention of a facilitator.

## Appendix 1 (CoP projects and tools)

### Norway

#### Induct

http://www.inductsoftware.com

A software development company that has developed an Innovation platform that is being used by the Oslo University Hospital and the UK NHS.

### United States

#### Harvard

http://catalyst.harvard.edu

Harvard University is recognised as having leading research groups in health-related areas.

The Catalyst Harvard group creates challenges to find solutions to specific medical issues.

#### InnoCentive

http://www2.innocentive.com

This company launches a set of challenges defined by enterprises and institutions. It then gathers ideas to address these challenges from different respondents and ideas collected privately, assesses them and defines the winner or winners of the challenge.

#### National Academy of Engineering

http://www.engineeringchallenges.org

This institution searches across different websites (using open innovation) to identify the most important challenges for engineering today, and later they present a deliverable with the conclusions.

#### Idea connection

http://www.ideaconnection.com

This is an open innovation group that develops solutions to problems presented by various companies. Although defined as open innovation, it is more of a distributed innovation system.

#### IdeaScale

http://ideascale.com

This company offers a platform to which users bring their challenges and receive proposals from other users. A wide range of companies and organisations, including the USA Government, use this platform to address challenges (http://opengov.ideascale.com).

#### Qmarkets

http://innovation.qmarkets.net

This company has developed software for managing ideas through open innovation called Ideation 2.0.

#### Incent Solutions

http://www.incentsolutions.com

This is another USA-based company that develops software for Open Innovation. The IDS-Innovate product has been developed for the automotive industry. Its adaptation to new markets is being studied.

### SPAIN

#### ideas4all

http://es.ideas4all.com/

This is a website developed in Madrid, to collect ideas and challenges to be developed through open innovation. Open to all themes and content, it is one of the first groups to develop an open innovation platform in Spain.

#### Europes World

http://www.europesworld.org

This was developed by the Catalonian government as a community of ideas at the European level. It primarily works with social issues.

#### Innova Health

http://www.saludinnova.com

This is a bank of innovative practices developed by the Andalusian Government. Users introduce ideas, knowledge about specific topics and best practices on the platform that may be of interest for other users in relation to innovation. The way it is being used, it could be considered as a tool for sharing knowledge.

#### Foro de inspiración

http://www.burubelarri.net/index.php

This is a space created for users, patients and health professionals, in order to bring forward new ideas to improve primary healthcare service in health centres in the district of San Sebastian.

## Abbreviations

CoP: Community of practice; EMR: Electronic medical record; GAM: Galder Abos Mendizabal; IZG: Irune Zaballa Gonzalez; RNS: Roberto Nuño Solinís; VCoP: Virtual community of practice; N/A: Not applicable.

## Competing interests

None of the authors have personal financial interests related to the subject matter discussed in the manuscript. HOBE + is an online professional community of practice to foster innovation in healthcare-related practice whose outputs are used to improve day-to-day practice in Basque Health Service. In this case, the innovation provider and the potential client are the same entity.

However, there is a potential conflict of interest as the paper’s authors play an active role in the day-to-day activity of HOBE+. More specifically:

GAM has lead and coordinated the HOBE + initiative since its creation, and continues to oversee the daily running of the process itself. He coordinates the work of the Ideas, Innovation and Management Groups and supports every stage of the life cycle of each idea selected by the Ideas Group.

RNS contributed with theoretical support at the time of creating HOBE + and validating O + berri’s role in the innovation business process.

IZG manages the Ideas Group idea filtering sessions.

## Authors’ contributions

GAM is the main contributor to this manuscript based on his experience and knowledge of HOBE + from its creation to the present day. RNS was also involved in drafting and revising the manuscript. IZG conducted the planning, execution and data analysis as well as collection of the survey data. All authors read and approved the final manuscript.

## Authors’ information

Galder Abos Mendizabal:

Senior Researcher at the Basque Institute for Healthcare Innovation (O + berri).

Honours Bachelor’s Degree in Industrial Organisation Engineering, University of Deusto.

Master’s Degree in Healthcare Management, Deusto Business School.

Roberto Nuño Solinís:

Director of the Basque Institute for Healthcare Innovation (O + berri).

Bachelor’s Degree in Economics and Business Administration, University of Deusto.

Postgraduate Degree in Health Economics, University of Tromsø.

Irune Zaballa González:

Junior Researcher at the Basque Institute for Healthcare Innovation (O + berri).

Bachelor’s Degree in Economics and Business Administration, University of the Basque Country (UPV-EHU).

## Pre-publication history

The pre-publication history for this paper can be accessed here:

http://www.biomedcentral.com/1471-2296/14/168/prepub

## References

[B1] Dahlander L Magnusson MG Relationships between open source software companies and communities Res Policy 2005 34 4 481 491 10.1016/j.respol.2005.02.003

[B2] Chesbrough HW Chesbrough HW, Vanhaverbeke W, West J New puzzles and new findings Open Innovation: Researching a new paradigm 2006 Oxford: Oxford University Press 15 34

[B3] Grant R Prospering in dynamically-competitive environments: Organizational capability as knowledge integration Organ Sci 1996 7 4 375 387 10.1287/orsc.7.4.375

[B4] Nonaka I The knowledge-creating company Harv Bus Rev 1991 68 96 104

[B5] Sheffield J Inquiry in health knowledge management J Knowl Manag 2008 12 160 172 10.1108/13673270810884327

[B6] Sáez Vacas F García O Palao J Rojo P Capitulo 14: Capital humano (y II): Gestión del conocimiento, e-Learning y modelos sociotécnicos Temas básicos de innovación tecnológica en las empresas 2003: Universidad Politécnica de Madrid

[B7] Nonaka I Takeuchi H The knowledge-creating company: How Japanese companies create the dynamics of innovation 1995 New York: Oxford University Press

[B8] Lave J Wenger E Situated Learning: Legitimate Peripheral Participation 1991 Cambridge: Cambridge University Press

[B9] Brown JS Duguid P Organizational learning and communities-of-practice: Toward a unified view of working, learning and innovation Organ Sci 1991 2 40 57 10.1287/orsc.2.1.40

[B10] Wenger E Communities of practice: learning, meaning and identity 1998 Cambridge: Cambridge University Press

[B11] Kislov R Harvey G Walshe K Collaborations for Leadership in Applied Health Research and Care: lessons from the theory of communities of practice Implement Sci 2011 6 64 10.1186/1748-5908-6-64 21699712 PMC3130688

[B12] Wenger E McDermott RA Snyder W Cultivating communities of practice: a guide to managing knowledge 2002 Boston: Harvard Business Press

[B13] Li LC Grimshaw JM Nielsen C Judd M Coyte PC Graham ID Evolution of Wenger's concept of community of practice Implement Sci 2009 4 11 10.1186/1748-5908-4-11 19250556 PMC2654669

[B14] Li LC Grimshaw JM Nielsen C Judd M Coyte PC Graham ID Use of communities of practice in business and health care sectors: A systematic review Implement Sci 2009 4 27 10.1186/1748-5908-4-27 19445723 PMC2694761

[B15] Barnett SR Jones SC Bennett S Iverson D Bonney A General practice training and virtual communities of practice - a review of the literature BMC Fam Pract 2012 13 87 10.1186/1471-2296-13-87 22905827 PMC3515460

[B16] Ranmuthugala G Plumb JJ Cunningham FC Georgiou A Westbrook JI Braithwaite J How and why are communities of practice established in the healthcare sector? A systematic review of the literature BMC Health Serv Res 2011 11 273 10.1186/1472-6963-11-273 21999305 PMC3219728

[B17] Le May A Communities of Practice in Health and Social Care 2009 Oxford: Wiley-Blackwell

[B18] Ranmuthugala G Plumb JJ Cunningham FC Georgiou A Westbrook JI Braithwaite J Communities of Practice in the health sector: a systematic review. [online monograph] 2010 Australia: Australian Institute of Health Innovation [Accessed 18 October 2012]. Available at: http://www3.chi.unsw.edu.au/pubs/CoP%20monograph.pdf

[B19] Lai KW Pratt K Anderson M Stigte JK Literature Review and Synthesis: Online Communities of Practice. [online monograph] 2006 New Zealand: Ministry of Education Report [Accessed 18 October 2012]. Available at: http://edcounts.squiz.net.nz/data/assets/pdf_file/0019/7480/lrs-online-com.pdf

[B20] Yin RK Case study research. Design and methods 2003 3 London: SAGE Publications

[B21] Probst G Borzillo S Why communities of practice succeed and why they fail Eur Manage J 2008 26 5 335 357 10.1016/j.emj.2008.05.003

[B22] Kislov R Walshe K Harvey G Managing boundaries in primary care service improvement: A developmental approach to communities of practice Implement Sci 2012 7 1 97 10.1186/1748-5908-7-97 23068016 PMC3514317

[B23] Rogers E Diffusion of Innovations 1962 Glencoe: Free Press 0-612-62843-4

[B24] Ferlie E Fitzgerald L Wood M Hawkins C The nonspread of innovations: The mediating role of professionals Acad Manage J 2005 48 117 134 10.5465/AMJ.2005.15993150

[B25] Gabbay J le May A Practice-Based Evidence for Healthcare: Clinical Mindlines 2011 Oxon: Routledge

